# Discovering the Individualized Factors Associated with Sarcopenia and Sarcopenic Obesity Phenotypes—A Machine Learning Approach

**DOI:** 10.3390/nu15214536

**Published:** 2023-10-26

**Authors:** Alessia Moroni, Simone Perna, Domenico Azzolino, Clara Gasparri, Roberta Zupo, Margherita Micheletti Cremasco, Mariangela Rondanelli

**Affiliations:** 1 Endocrinology and Nutrition Unit, Azienda di Servizi Alla Persona “Istituto Santa Margherita”, University of Pavia, 27100 Pavia, Italy; clara.gasparri01@universitadipavia.it; 2 Department of Food, Environmental and Nutritional Sciences, Division of Human Nutrition, University of Milan, 20133 Milan, Italy; 3 Geriatric Unit, Fondazione IRCCS Ca’ Granda Ospedale Maggiore Policlinico di Milano, 20122 Milan, Italy; 4 Department of Interdisciplinary Medicine (DIM), University “Aldo Moro”, Piazza Giulio Cesare 11, 70100 Bari, Italy; roberta.zupo@uniba.it; 5 Department of Life Science and Systems Biology, University of Torino, Via Accademia Albertina, 13, 10123 Torino, Italy; margherita.micheletti@unito.it; 6 Department of Public Health, Experimental and Forensic Medicine, University of Pavia, 27100 Pavia, Italy; mariangela.rondanelli@unipv.it

**Keywords:** sarcopenia, sarcopenic obesity, osteosarcopenia, osteosarcopenic obesity, importance analysis

## Abstract

The literature shows how sarcopenia often occurs along with different phenotypes based either on the concomitant presence of adipose tissue excess (i.e., sarcopenic obesity, SO), or osteopenia/osteoporosis (osteosarcopenia, OS), or the combination of the two conditions, so-called osteosarcopenic obesity (OSO). This research aimed to assess the prevalence of sarcopenia phenotypes (SO, OS, OSO), their associated risk factors and their health impact in a population of out- and inpatients living in the North of Italy. Male and female subjects aged ≥18 years were enrolled for the study. A blood sample was collected to measure targeted blood makers. A comprehensive anthropometric clinical assessment (height, weight, Body Mass Index, BMI and Dual Energy X-ray Absorptiometry, DXA) was performed to measure ponderal, bone, fat, and muscle status. A total of 1510 individuals participated to the study (females, n = 1100; 72.85%). Sarcopenia was the most prevalent phenotype (17%), followed by osteosarcopenia (14.7%) and sarcopenic obesity. Only 1.9% of the sample was affected by OSO. According to logistic regression analysis, sarcopenia was associated with age, erythrocyte sedimentation rate (ESR), C-reactive protein (CRP) (positively) and BMI, Iron (Fe), Total Cholesterol, albumin (%), albumin (g), and gamma proteins (negatively). Sarcopenic obesity was associated with age, ferritin, ESR, CRP (positively) and BMI, Fe, and albumin (%) (negatively). Osteosarcopenia was associated with age, ESR (positively) and BMI, Total Cholesterol, albumin (%), albumin (g), and Ca (negatively). Osteosarcopenic obesity was associated with glycemia and gamma-glutamyl transferase (gGT) (positively). According to random forest analysis, a higher BMI was the most important protective factor for sarcopenia, for sarcopenic obesity (along with Iron) and for osteosarcopenia (along with albumin). Moreover, osteosarcopenic obesity was positively associated with GgT and glycaemia. The possibility of gaining such information, especially in the younger population, could help to prevent the onset of such diseases and best fit the patient’s needs, according to a precision-medicine approach.

## 1. Introduction

According to the latest guidelines by the European Working Group on Sarcopenia in Older People (EGSWOP2) [1], sarcopenia is a disease which involves the decline of both muscular strength and muscle mass, while severity is determined by low physical performance. Particularly, evidence shows how sarcopenia often occurs along with different phenotypes based on the presence of adipose tissue excess (sarcopenic obesity, SO, [2]), of osteopenia/osteoporosis diagnosis (osteosarcopenia, OS, [3]), or even of the combination of the three conditions, resulting in osteosarcopenic obesity (OSO) [4]. 

More than 50 million people worldwide experience sarcopenia, and it is estimated that over 200 million people will suffer sarcopenia in the next four decades [5]. 

The prevalence of the associated phenotypes (SO, OS, OSO) depends on the study cohort, age, and comorbidities, as well as on diagnosis criteria, which are not the same worldwide. However, a recent systematic review and meta-analysis estimated that, in middle-aged and older adults, the global prevalence of osteosarcopenic obesity, which indicates the combination of all the considered phenotypes, was 8%, with it affecting women (9%) more than men (5%) [6]. 

Sarcopenia and osteoporosis share some risk factors and the same biological molecular pathways, underlying the onset of the disease stage [7,8]. Beyond genetic factors, which seem to play a preponderant role in the determination of both diseases, ageing has a significant impact, along with lifestyle habits such as sedentary behaviour and nutrition. Indeed, during ageing, metabolism and body composition slowly change, resulting in a decrease in basal metabolic rate, reflecting the decrement of tissues’ metabolic activity and reduced energy consumption [9]. With ageing, adipose tissue increases along with fat infiltration of the muscle, and it is associated with the loss of bone mass, while myosteatosis is linked with a loss of myofiber function and the subsequent decrease in muscle mass. Additionally, the alterations of adipose tissue functions during ageing yields a high amount of inflammatory peptides, thus resulting in an increased infiltrate of inflammatory cells (inflammageing, a low-grade chronic inflammation which involves the constant stimulation of the immune system) [10].

In a recent study, Zupo et al. (2023) [11] found that the predicted risk factors for the development of sarcopenia seemed to be albumin, C-reactive protein (CRP), folate, and ageing, according to Random Forest selection, while gender, folate, and vitamin D deficiency were the most relevant according to logistics. Campos et al. (2020) [12] found that sarcopenic obesity was associated with CRP, glucose, albumin and insulin resistance. For what concerns osteosarcopenia, Inoue et al. (2021) and Kaji et al. (2014) [13,14] explained how osteopenia/osteoporosis and sarcopenia have common risk factors such as ageing, gender, inactivity, reduced vitamin D, growth hormone, insulin-like growth factor I and testosterone. The authors found cross-sectional associations with age, sex, frailty, chronic disease, physical function, nutrition, and the endocrine system, which were found to be a risk factor for fractures and falls. Additionally, osteosarcopenic obesity has been associated with low vitamin D levels, hypertension and dyslipidaemia [15,16,17,18,19,20], as well as with adverse outcomes in terms of morbidity, mortality and Quality of Life (QoL) in several domains [21,22]. 

The aim of this study was to assess the prevalence of sarcopenia phenotypes (SO, OS, OSO), their associated risk factors and, subsequently, their health impact in a population of out- and inpatients living in the North of Italy (Lombardia).

## 2. Materials and Methods

### 2.1. Study Population

We enrolled male and female subjects, aged ≥18 years, attending the metabolic rehabilitation unit of the Santa Margherita Institute, Department of Public Health, University of Pavia. Subjects with severe pathologies were excluded from the study. The recruitment period was between January 2011 and January 2023. Informed consent was obtained from participants. 

The study was conducted according to the guidelines of the Declaration of Helsinki and was approved by the Ethics Committee of the University of Pavia (ethical code Number: 2109/14022019).

### 2.2. Anthropometric and Clinical Assessment

Height was measured to the nearest 0.5 cm using a wall-mounted stadiometer (Mod C 201, Wunder SA.BI. Srl). Body weight was determined at the time of DXA to the nearest 0.1 kg using a calibrated balance beam scale (Mod C 201, Wunder SA.BI Srl). BMI was calculated as weight in kilograms divided by height in metres squared (kg/m^2^). Bone mineral density (BMD) and whole-body lean mass were measured using DXA (Lunar Prodigy DXA; GE Healthcare Medical Systems, Chicago, IL, USA) together with the DXA Prodigy encore software (version 17; GE Healthcare). The skeletal muscle mass index (SMI) was defined as the sum of the muscle masses of the four limbs, as appendicular skeletal muscle mass, divided by height squared. Whole-body lean mass (kg) was taken as the sum of the fat-free, bone-free mass of the arms and legs as the lean mass. 

A blood sample was collected in the morning after overnight fasting to measure the levels of fasting blood glucose (FBG), Total Cholesterol, high-density lipoprotein (HDL) cholesterol, low-density lipoprotein (LDL) cholesterol, triglycerides, Fe, Ferritin, albumin (g and %), gamma proteins, GgT, serum tri-iodothyronine (FT3), thyroxine (FT4), thyroid stimulating hormone (TSH), serum high-sensitivity C-reactive protein (CRP), their erythrocyte sedimentation rate (ESR), calcium and serum 25(OH) vitamin D.

Despite the awareness of the newly published criteria for the diagnosis of sarcopenia and sarcopenic obesity [1,23], the cut-offs described by Kelly et al. (2019) [24], which are specific for the diagnosis of osteosarcopenic obesity, were used in order to homogenize the criteria for the individualization of each phenotype. Low muscle mass was defined by the presence of low muscle mass (i.e., SMI < 7.26 kg/m^2^ for men or <5.45 kg/m^2^ for women), while sarcopenic obesity was defined if sarcopenia was accompanied by >25% of total body fat for men and >32% for women. Osteosarcopenia was defined as the concomitant presence of sarcopenia and osteopenia (≤−1.0)/osteoporosis (≤2.5 SD) (T-score for Bone Mineral Density [BMD] at the femoral neck ≤ −1.0 standard deviation [SD]). As we could not assess visceral fat using computed tomography (CT) or Magnetic Resonance Imaging (MRI) but using DXA, we used the cut-off presented by Lundblad et al. (2021) [25], comparing them in relation to VAT cut-offs proposed by Kelly et al. (2019) [24]. Indeed, we adapted DXA obtained VAT values in g, using the Lundblad et al. (2021) cut-off (men VAT: >1859 g and women: >1134 g) [25]. 

### 2.3. Data Analysis

The sample was primarily divided into three age categories, according to Von Humboldt et al. (2015) [26]: Adult (<65 years old); Oldest (age from 66 to 84); and Oldest old (≥85 years old). IBM SPSS software (version 28) was used to perform statistical analysis. Normality of data was assessed using the Kolmogorov–Smirnov test; the majority of variables was not normally distributed, so we used a non-parametric approach. Statistical significance was accepted for *p* < 0.05. Descriptive statistics were reported as the mean ± standard deviation (SD) for continuous measures and frequency and percentages (%) for all categorical variables. Logistic regressions were performed dividing regressors into clusters, depending on blood profile groups, which were gender (1), age and BMI (2) Iron status (3) (Fe, ferritin), lipid profile (4) (Total Cholesterol, HDL), protein profile (5) (albumin %, albumin g, gamma proteins), thyroid functionality (6) (THS, FT3, FT4), inflammation (7) (ESR, CRP), micronutrients (8) (calcium, vitamin D 25(OH)), GgT (9) and glycemia (10). Subsequently, only for the variables that were definitely associated, researchers performed a Neural Networks analysis in order to assess the impact of the variable on the outcome and, thus, the importance in terms of accuracy.

## 3. Results

### 3.1. The Sample

Overall, 1510 individuals participated in the study protocol (women, n = 1100; 72.85%). Age and BMI descriptive statistics are presented in Table 1, while the prevalence of sarcopenia, sarcopenic obesity, osteosarcopenia and osteosarcopenic obesity is reported in Table 2. Considering the whole sample in terms of mean age, the majority of the population pertained to the oldest age category, even though the SD was relatively high. The mean BMI highlighted the overweight condition of the overall sample, but this information could be misinterpreted considering the very high SD, which pointed out the ponderal heterogeneity of the sample. Among the targeted diseases, sarcopenia was the most prevalent phenotype (17% of the entire sample), followed by osteosarcopenia (14.7%) and sarcopenic obesity. Only 1.9% of the sample was found to be affected by osteosarcopenic obesity. Overall, sarcopenia-related phenotypes were much greater in the male samples compared to the female counterpart, as shown in Table 2.

### 3.2. Logistic Regression Analysis

The results of the regression analysis are presented in Table 3.

#### 3.2.1. Gender

All the phenotypes were found to be negatively associated with sex; this means that males have more risk of developing the diseases investigated (*p* < 0.001 for all the cases). For all the cases, females had lower risk (from 69.1 to 79.9%) for the onset of the conditions.

#### 3.2.2. Age and BMI

Three out of the four sarcopenia phenotypes were associated with age and BMI. Concerning age, it was found to be positively associated with sarcopenia and osteosarcopenia (*p* < 0.001) and less, but still significantly so, with sarcopenic obesity (*p* < 0.05). The risk increases by a few units, with the highest value for osteosarcopenia. BMI was negatively associated with sarcopenia, sarcopenic obesity and osteosarcopenia (*p* < 0.001). With the increase in BMI, the risk of the onset of sarcopenia and osteosarcopenia decreases by more than 20%.

#### 3.2.3. Iron and Haematocrit Status

Sarcopenia and sarcopenic obesity were negatively associated with Fe (*p* < 0.05 and *p* < 0.01, respectively), meaning that the increase in Fe is a protective factor for the onset of such diseases. Conversely, sarcopenic obesity was also positively associated with ferritin (*p* < 0.05), even though the risk percentage was almost irrelevant.

#### 3.2.4. Lipid Profile

Regarding the lipid profile, sarcopenia and osteosarcopenia were negatively associated with Total Cholesterol (*p* < 0.05 and *p* < 0.01, respectively), indicating that an increase in Total Cholesterol seems to be a protective factor.

#### 3.2.5. Protein Profile

Sarcopenia, sarcopenic obesity and osteosarcopenia were negatively associated with albumin (%) (*p* < 0.001 for sarcopenia and osteosarcopenia, and *p* < 0.01 for sarcopenic obesity). Sarcopenia and Osteosarcopenia were also negatively associated with albumin (g) (*p* < 0.01; *p* < 0.05, respectively). Moreover, sarcopenia was also negatively associated with gamma proteins.

#### 3.2.6. Inflammation Profile

For what concerns inflammation, sarcopenia, sarcopenic obesity and osteosarcopenia were positively associated with ESR (*p* < 0.01, *p* < 0.05 and *p* < 0.05, respectively). Sarcopenia and sarcopenic obesity were also positively associated with CRP (*p* < 0.05 for both cases). The risk of developing the disease is much greater when the unit increase occurs for CRP, while the risk percentage concerning ESR is almost irrelevant.

#### 3.2.7. Association with Serum Biomarkers

Osteosarcopenia was negatively associated with Ca (*p* < 0.05), making it a protective factor. Osteosarcopenic obesity was positively associated with GgT (*p* < 0.01) and with glycemia (*p* < 0.01).

In summary, sarcopenia was associated with age, ESR, RCP (positively) and BMI, Fe, Total Cholesterol, albumin (%) albumin (g), and gamma proteins (negatively). Sarcopenic obesity was associated with age, ferritin, ESR, CRP (positively) and BMI, Fe, and albumin (%) (negatively). Osteosarcopenia was associated with age, ESR (positively) and BMI, Total Cholesterol, albumin (%), albumin (g), and Ca (negatively). Osteosarcopenic obesity was associated with glycemia and gGT (positively).

### 3.3. Neural Network Analysis

In order to understand the effective impact of the associated fluid biomarkers, we performed an analysis of importance (i.e., Neural Network analysis or Random Forest) for each sarcopenia phenotype.

#### 3.3.1. Sarcopenia

**Figure 1:**
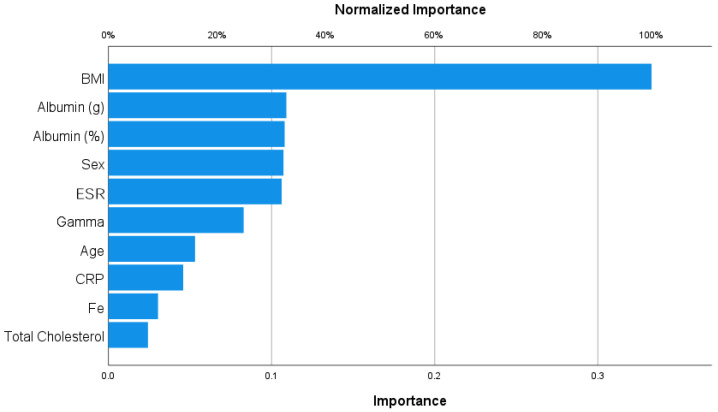
Importance analysis for sarcopenia.

As shown in Figure 1, the only important variable was BMI (importance = 0.333; normalized importance 100%). As shown in the logistic regression analysis, it was a protective factor.

#### 3.3.2. Sarcopenic Obesity

**Figure 2:**
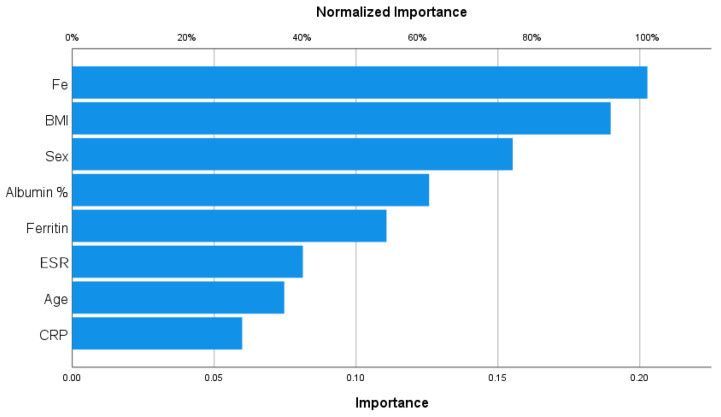
Importance analysis for sarcopenic obesity.

With regard to sarcopenic obesity (Figure 2), Fe and BMI were the most important variables and were protective factors (importance = 0.203, 0.190, respectively; normalized importance, 100%; 93.6, respectively).

#### 3.3.3. Osteosarcopenia

**Figure 3:**
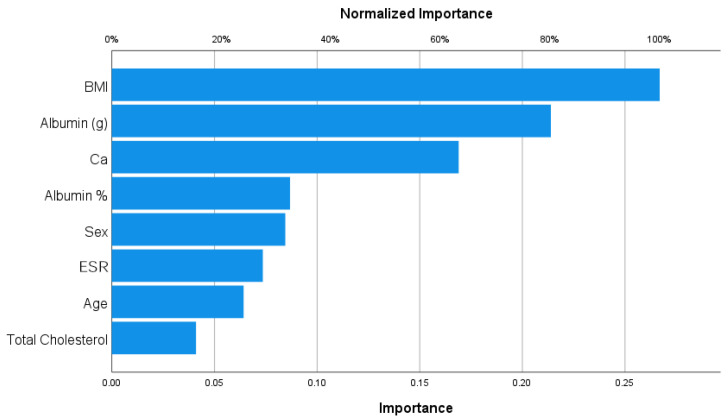
Importance analysis for osteosarcopenia.

Body Mass Index (BMI) and albumin (g) were the most important variables and were protective factors to osteosarcopenia, as shown in Figure 3 (importance = 0.267, 0.214, respectively; normalized importance, 100%, 80.1, respectively).

#### 3.3.4. Osteosarcopenic Obesity

**Figure 4:**
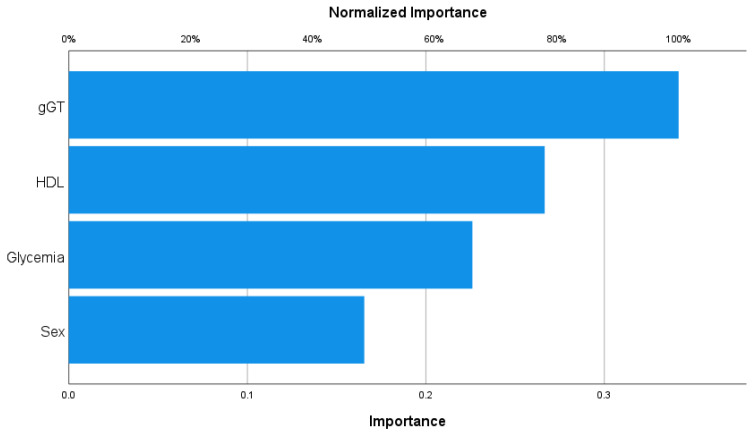
Importance analysis for osteosarcopenic obesity.

Concerning osteosarcopenic obesity (Figure 4), among all the associated variables, gGT was the most impactful factor on this phenotype (importance = 0.342; normalized importance, 100%).

## 4. Discussion

The aim of this study was to assess the prevalence of sarcopenia phenotypes (SO, OS, OSO), as well as its associated risk factors, in a population of out- and inpatients living in the North of Italy (Lombardia). Moreover, the researchers aimed to also investigate the impact of such risk factors in terms of health impact.

The considered population was mainly composed of patients between 66 and 84 years old, even though it was possible to also include younger (Adult) and older (Oldest Old) people. Considering BMI, although it had a high SD, it was possible to observe how the ponderal status trend tended to reach lower values in older people, while the younger population was overweight or even obese. Indeed, the Adult group was mainly obese, the Oldest category was still overweight, and the Oldest Old was normoweight. Despite this study using a cross-sectional approach, such data suggested that during the last stage of ageing, it is more likely that people will tend to decrease their BMI, generally due to a consistent loss in skeletal muscle mass caused by a decrease in nutrition intake (particularly protein intake) and less movement.

The epidemiological overview of the sarcopenia phenotypes evaluated in this study could not be compared with previous studies, as the literature concerning the combination of the four phenotypes, as well as osteosarcopenic obesity, is lacking. To our knowledge, so far only Perna et al. (2018) [27] have investigated all the above-mentioned phenotypes, in a study conducted in 480 inpatients. In that research, the authors highlighted how the rate of osteosarcopenic obesity was much higher than the percentage found in this study (1.9%), as they reported 6.86% of people affected. It is tough to underline the difference in terms of the sample size and age between their population and the present one, as well as the different inclusion criteria between the methods. A recent systematic review and meta-analysis concerning osteosarcopenic obesity reported how such a condition’s prevalence ranged from 1.5 to 65.7% and impacted females more than males, as their Bone Mineral Density (BMD) is generally lower and the changes in oestrogen levels in females can affect the functions of bones and muscles [28]. The OSO prevalence found in our study (1.9%) was, thus, in line with the range considered in the study of Huang et al. (2023) [28] but, conversely, males were always the most affected population for all the phenotypes. Indeed, sarcopenia was the most prevalent phenotype, while only 1.9% of the sample was affected by osteosarcopenic obesity.

Due to the heterogeneity of data presented in the literature in relation to sarcopenia phenotypes, it is very difficult to compare our results with previous research, although it was still possible for some variables. Firstly, our results converged with others concerning the fact that males are at higher risk of developing sarcopenia and related phenotypes, probably due to the physiological path of the reduction in testosterone [11,29].

Moreover, age was an important risk factor for the development of sarcopenia, sarcopenic obesity and osteosarcopenia, but not for osteosarcopenic obesity. Indeed, it is well known that ageing affects mostly BMD and skeletal muscle mass, leading to a loss of muscle strength and mass and subsequently changing the skeletal microstructure and decreasing mineral density, resulting in decreased bone mass [28,30]. BMI was observed to be one of the most important variables, as it was a protective factor of sarcopenia, sarcopenic obesity and osteosarcopenia, and, according to the random forest analysis, it was also the most important related factor for sarcopenia, sarcopenic obesity (along with Fe) and for osteosarcopenia (along with albumin). Indeed, some studies have reported how a higher BMI could be actually protective for the onset of sarcopenia, as individuals with higher fat mass might have a higher nutritional intake (in particular protein intake) as well as lipid storage and can, thus, preserve their normal weight during the delicate phase of ageing [11,31].

Iron and Iron accumulation (indicated by ferritin) seem to be associated with sarcopenic obesity. As Fe is considered an important and protective factor, ferritin was found to be positively associated with such a phenotype (even tough very slightly) and could, thus, reflect the actions of excess iron, affecting insulin synthesis and secretion in the pancreas and interfering with the insulin-extracting capacity of the liver, thereby leading to peripheral hyperinsulinemia and impaired insulin secretion [32,33].

Along with BMI, albumin was the most important factor associated with osteosarcopenia, indicating how a balanced protein profile is crucial for preventing the onset of both osteoporosis and sarcopenia [34].

Interestingly, GgT was the most important factor for osteosarcopenic obesity, showing a positive relation similar to that found for glycaemia, meaning they could be associated with the development of OSO. Despite this, such results must be interpreted with caution, especially considering the very low percentage of risk (0.9%).

This study had some limitations: firstly, the majority of the sample was composed of females, and the age subgroups were not homogeneous as the most consistent category was the Oldest one. Additionally, in order to homogenize the criteria for the individualization of each sarcopenia phenotype, we used the cut-off described by Kelly et al. (2019) [24], which does not take Handgrip Strength into account. Moreover, this study was conducted in a specific setting so the population could not be representative, and results can, thus, not be generalized. It would be then desirable to conduct multicentric studies that involve a wider population, homogeneous for gender and age category, to confirm our results.

## 5. Conclusions

To our knowledge, this is one of the first studies investigating the prevalence of sarcopenia-related phenotypes, their related risk factors and their importance in a population-based sample from Northern Italy. We found that being male was considered a risk factor for all the phenotypes, while a higher BMI was the most important protective factor for sarcopenia, sarcopenic obesity (along with Fe) and osteosarcopenia (along with albumin). Moreover, osteosarcopenic obesity was positively associated with GgT and glycaemia. The possibility of gaining such information, especially in a younger population, could help to prevent the onset of such diseases and best fit a patient’s needs, according to a precision medicine approach.

## Figures and Tables

**Table 1:** Descriptive statistics in the considered population. Subgroups depending on age (Adult < 65 years; Oldest 66–84 years; Oldest Old > 84 years) were made according to Van Humboldt et al. (2015) [26].

	Whole Sample (n = 1510)	Adult (n = 198)		Oldest (n = 1019)		Oldest Old (n = 293)		*p* Value Males	*p* Value Females
		Males (n = 47) (mean ± SD)	Females (n = 151) (mean ± SD)	Males (n = 309) (mean ± SD)	Females (n = 710) (mean ± SD)	Males (n = 54) (mean ± SD)	Females (n = 239) (mean ± SD)		
Age (years)	75.91 ± 12.89	49.96 ± 13.56	50.23 ± 12.50	76.61 ± 5.71	77.23 ± 5.42	88.78 ± 2.37	89.48 ± 2.79	<0.001	<0.001
BMI (kg/m^2^)	26.25 ± 8.37	32.59 ± 8.98	30.29 ± 18.53	25.68 ± 4.84	26.31 ± 6.59	22.97 ± 3.71	23.75 ± 4.64	<0.001	<0.001
Fe (mcg/dL)	65.94 ± 32.81	69.55 ± 26.46	81.60 ± 45.27	68.41 ± 37.02	67.75 ± 32.18	58.64 ± 24.59	59.15 ± 29.20	0.30	<0.01
Ferritin (ng/mL)	176.14 ± 159.39	159.50 ± 129.49	107.63 ± 78.58	271.06 ± 304.39	146.71 ± 280.50	240.07 ± 156.54	132.66 ± 104.56	0.66	0.82
Total Cholesterol (mg/dL)	183.52 ± 44.15	166.18 ± 49.14	197.57 ± 47.70	168.91 ± 38.44	192.1 ± 44.40	163.61 ± 40.24	183.83 ± 44.15	0.73	0.1
Albumin (g)	3.83 ± 3.51	3.67 ± 0.44	4.00 ± 0.31	3.63 ± 0.56	3.69 ± 0.45	4.91 ± 9.43	4.13 ± 5.99	0.18	0.32
Albumin (%)	54.97 ± 6.24	57.44 ± 5.37	57.51 ± 4.37	54.95 ± 6.38	55.51 ± 5.47	52.39 ± 9.74	53.97 ± 6.62	<0.05	<0.01
Gamma Protein (g/L)	16.80 ± 4.58	14.83 ± 3.01	17.14 ± 5.35	17.44 ± 4.88	16.33 ± 4.54	17.26 ± 4.08	17.22 ± 4.40	0.18	0.09
ESR (mm/hr)	43.55 ± 31.19	24.58 ± 24.17	37.53 ± 27.78	41.44 ± 31.67	43.20 ± 30.55	41.18 ± 29.22	48.97 ± 32.72	0.19	0.10
CRP (mg/dL)	1.29 ± 2.70	0.94 ± 1.07	0.81 ± 1.91	1.74 ± 3.51	1.07 ± 2.36	1.85 ± 3.29	1.29 ± 2.40	0.71	0.51
Ca (mmol/L)	10.08 ± 27.68	9.05 ± 0.56	9.53 ± 0.59	9.12 ± 0.90	9.20 ± 0.61	8.67 ± 1.44	13.50 ± 60.17	<0.05	0.34
gGT (U/L)	32.27 ± 37.91	28.58 ± 13.93	29.40 ± 20.51	34.09 ± 34.35	32.36 ± 42.49	30.10 ± 24.38	31.31 ± 34.99	0.69	0.93
Glycemia (mg/dL)	107.58 ± 41.15	96.77 ± 30.58	133.07 ± 80.79	111.83 ± 44.66	106.78 ± 40.09	109.60 ± 38.50	103.11 ± 37.73	0.44	<0.05

**Table 2:** Prevalence of sarcopenia and related phenotypes (SO, OS, OSO) in the considered population. Subgroups (Adult < 65 years; Oldest 66–84 years; Oldest Old > 84 years) depending on age were made according to Van Humboldt et al. (2015) [26].

	Whole Sample (n = 1510)	Adult (n = 198)		Oldest (n = 1019)		Oldest Old (n = 293)	
		Males (n = 47) N (%)	Females (n = 151) N (%)	Males (n = 309) N (%)	Females (n = 710) N (%)	Males (n = 54) N (%)	Females (n = 239) N (%)
Sarcopenia	264 (17.5%)	8 (17%)	2 (1.3%)	109 (35.3%)	73 (10.3%)	29 (53.7%)	43 (18%)
Sarcopenic Obesity	159 (10.5%)	6 (12.8%)	2 (1.3%)	71 (23%)	43 (6.1%)	17 (31.5%)	20 (8.4%)
Osteosarcopenia	222 (14.7%)	5 (10.6%)	2 (1.3%)	84 (27.2%)	68 (9.6%)	21 (38.9%)	42 (17.6%)
Osteosarcopenic Obesity	29 (1.9%)	2 (4.3%)	0 (0%)	14 (4.5%)	8 (1.1%)	1 (1.9%)	4 (1.7%)

**Table 3:** Logistic regression analysis.

		B (Regression’s Coefficient)	*p* Value	Odds Ratio	Risk
Gender Females (reference category Males)					
	Sarcopenia	−1.527	<0.001	0.217	−78.3%
	Sarcopenic obesity	−1.555	<0.001	0.211	−79.9%
	Osteosarcopenia	−1.173	<0.001	0.309	−69.1%
	Osteosarcopenic obesity	−1.367	<0.001	0.255	−74.5%
Age and BMI					
Age (from lowest to highest)					
	Sarcopenia	0.027	<0.001	1.027	2.7%
	Sarcopenic obesity	0.016	<0.05	1.017	1.7%
	Osteosarcopenia	0.031	<0.001	1.031	3.1%
BMI (from lowest to highest)					
	Sarcopenia	−0.282	<0.001	0.754	−24.6%
	Sarcopenic obesity	−0.123	<0.001	0.884	−11.6%
	Osteosarcopenia	−0.272	<0.001	0.762	−23.8%
Iron and haematocrit status					
Fe (from lowest to highest)					
	Sarcopenia	−0.014	<0.05	0.986	−1.4%
	Sarcopenic obesity	−0.024	<0.01	0.976	−2.4%
Ferritin (from lowest to highest)					
	Sarcopenic obesity	0.001	<0.05	1.001	0.1%
Lipid profile					
Total Cholesterol (from lowest to highest)					
	Sarcopenia	−0.026	<0.05	0.974	−2.6%
	Osteosarcopenia	−0.032	<0.01	0.969	−3.1%
Protein Profile					
Albumin (%) (from lowest to highest)	Sarcopenia	−0.135	<0.001	0.874	−12.6%
	Sarcopenic obesity	−0.130	<0.01	0.878	−12.2%
	Osteosarcopenia	−0.127	<0.001	0.881	−11.9%
Albumin (g) (from lowest to highest)					
	Sarcopenia	−0.102	<0.01	0.903	−9.7%
	Osteosarcopenia	−0.089	<0.05	0.915	−8.5%
Gamma proteins (from lowest to highest)					
	Sarcopenia	−0.074	<0.05	0.929	−7.1%
Inflammation					
ESR (from lowest to highest)					
	Sarcopenia	0.008	<0.01	1.008	0.8%
	Sarcopenic obesity	0.007	<0.05	1.007	0.7%
	Osteosarcopenia	0.008	<0.05	1.008	0.8%
CRP (from lowest to highest)					
	Sarcopenia	0.072	<0.05	1.074	7.4%
	Sarcopenic obesity	0.082	<0.05	1.085	8.5%
Micronutrients					
Ca (from lowest to highest)					
	Osteosarcopenia	−0.449	<0.05	0.638	−36.2%
Other					
GgT (from lowest to highest)					
	Osteosarcopenic obesity	0.009	<0.01	1.009	0.9%
Glycemia (from lowest to highest)					
	Osteosarcopenic obesity	0.009	<0.01	1.009	0.9%

## Data Availability

The data presented in this study are available on request from the corresponding authors. The data are not publicly available due to privacy reasons.
